# Supplementary material for: Are carbon emissions trading and green financial instruments synergistic? -Comprehensive quantitative research based on content analysis
Source: PLoS One. 2024 Mar 7;19(3):e0298601. doi: 10.1371/journal.pone.0298601 (PMC10919601; doi:10.1371/journal.pone.0298601)
Supplement: S1 Data — (ZIP) [file pone.0298601.s001.zip › Data/地级市Z_F工作报告.zip!/║╙─╧/╨┬╧τ╩╨/└┤╫╘╣1⁄2╓┌║┼í╛┬φ┐╦╩2╛▌═°í┐_2010.docx]

各位代表：

现在，我代表市人民政府向大会做工作报告，请予审议，并请各位政协委员和列席人员提出意见。

一、2009年工作回顾

2009年是近些年经济工作比较艰难的一年，在省委、省政府和市委的坚强领导下，在市人大、市政协的监督支持下，市政府团结带领全市人民，深入贯彻科学发展观，坚持“长短结合、结构调整、应对挑战、晋位升级”的思路，强化运行调节，加快项目建设，调整产业结构，努力改善民生，除进出口总额、农民人均纯收入增速外，各项主要工作均较好地完成了市十一届人大一次会议确定的任务。预计全年实现地区生产总值1054.6亿元，比上年增长（下同）13%，增速居全省第2位；财政一般预算收入55.9亿元、支出131.4亿元，分别增长14.6%和32.5%，收入总量由全省第7位上升到第5位；完成城镇固定资产投资904.5亿元，增长30.8%，总量稳居全省第4位；规模以上工业增加值430.2亿元，增长16.1%；粮食总产379万吨，大旱之年再创历史新高；年末金融机构各项存款余额941亿元，各项贷款余额621亿元，分别增长23.9%和22.7%；全社会消费品零售总额330.3亿元，增长19%；城镇居民人均可支配收入、农民人均纯收入分别达14170元和5431元，实际增长10.3%和7.6%。主要做了以下工作：

（一）强化服务，帮助企业度过难关。保证企业正常经营和持续发展是应对危机的关键所在。我市深入开展企业服务年活动，及时出台措施，强化煤电油气运及资金保障，引导企业战略重组，全力为企业排忧解难。共为1010户规模以上工业企业解决问题1296项，问题解决率居全省第一。组建30家信用担保机构，召开银企洽谈会12次，贷款到位资金117亿元。为企业减免税款18.4亿元。新增建设用地总量在全省名列前茅。受金融危机影响较重的停产半停产企业去年上半年全部恢复正常生产。全年工业用电量增长8.8%。

（二）坚持引资项目带动，增强发展后劲。抓住中央扩大投资的政策机遇，坚持走出去招商引资、大力争取上级资金和政策支持，一批重大项目先后落地并开工，跨越发展的态势得到巩固。招商工作成效明显。成功举办了市庆60周年项目推介会，在杭州、广州、厦门、福州、澳门等地多次进行招商推介，中粮集团、中航集团等中央企业和雨润集团、双鹭药业、北京圣宝运等知名企业投资项目落地。招商引资工作综合排名居全省第3位。实际利用外资2.53亿美元，增长12.3%，总量居全省第4位；实际利用市外资金168亿元，增长38.7％。进出口总额完成10.4亿美元。项目建设再创佳绩。新开工千万元以上项目1980个，同比增加511个，完成投资增长 44.5%；新开工亿元以上项目99个，同比增加33个，完成投资增长20.8%。省、市145个重点项目中，金龙集团年产6亿安时动力锂离子电池、中粮（新乡）小麦仓储等50个项目开工建设，赵固一矿年产240万吨原煤、心连心化肥合成氨尿素系列产品等45个项目竣工达产。在两次全省重点项目观摩中我市都被评为豫北片第1名。项目质量不断提升。工业投资占城镇固定资产投资的66.4%，占全省工业投资的9.4%。重点工业项目中高科技项目占40%。绿源药业等7个投资5亿元以上的结构调整项目竣工投产，双鹭生物新医药基地等结构升级重点项目开工建设。

（三）着力调整转型，优化经济结构。积极主动地调结构、促转型，经济发展质量和企业竞争力在逆境中得到提升。一、二、三产业的比例由上年的13.8：54.9：31.3调整为12.5：55.5：32。工业企业赢利能力增强。规模以上工业企业实现利润、利税分别增长38.4%和31.1%，分居全省第4位和第1位，获得国家、省淘汰落后产能奖励资金居全省第一。“一谷五基地”六大战略支撑产业总产值、利润分别占全市的62.1%和56.6%。矿山集团等9个战略重组项目完成，煤化工企业集团等5个战略重组项目积极推进，一批中央企业、知名外企与我市企业的战略合作不断拓展。83户市属企业改制任务基本完成。高效农业健康发展。农田水利基本建设扎实推进，获得省 “红旗渠精神杯”五连冠。种植业结构不断优化，新认定无公害农产品、绿色食品、有机食品和地理标志农产品基地面积25.8万亩，新认证产品35个。大力发展品牌农业、高端农业，太空米、迪一米、金粒小麦、宏力葡萄等名牌农产品走俏市场。新认定无公害畜产品10个，累计达33个，连续6年居全省第1位。畜牧业产值92.8亿元，占农业总产值的40.6%。农业产业化龙头企业425家，实现销售收入260亿元。农民专业合作社1175家，带动农户27.8万户。现代服务业加速发展。海宁皮革城、万惠城市综合体等项目落地，大商新玛特、平原路地下商业街等项目竣工。共发放家电、汽车摩托车下乡补贴9873万元。完成房地产开发投资102.2亿元，增长33.9%；商品房销售289.7万平方米，增长32.6%。国内外游客接待量、旅游综合收入分别增长21.4%和24.3%。节能减排扎实推进。全年化学需氧量、二氧化硫排放量分别控制在6.34万吨和5.7万吨，单位生产总值能耗降低4.6%，完成了年度工作目标。城市环境空气质量优良天数达标率93.8%，集中饮用水源地取水水质达标率保持100%。

（四）加快自主创新，助推产业升级。完善技术创新服务平台，推进产学研结合，依靠科技创新更好地应对危机。依托中国农科院、中科院和河南农科院在我市建立的现代农业示范基地以及驻新高校和科研机构，开展新品种、新技术研发，推进农科教一体化，现代农业立体科技支撑体系初步形成。80%的规模以上企业与科研院所、高校建立合作伙伴关系，省重大科技专项数量及其扶持资金、省知识产权优势企业个数均居全省第1位，中国名牌产品、省级名牌产品数量分居全省第4位和第2位。新认定国家高新技术企业10家，累计达到19家，居全省第3位。新组建省级企业研发中心8家，总数达94家，占全省十分之一。高新技术产业增加值比重达29％，申请专利1456项，均居全省前列。新乡县被确定为第三批国家科技进步示范县，长垣县被列入国家知识产权强县推进工程。市农科院“新麦18号”项目获得省科技进步一等奖，华兰生物甲型H1N1流感疫苗产品全国市场占有率接近40%，中铁隧道集团新乡公司研制的复合盾构机打破了国外技术垄断。

（五）推进城乡统筹，增添发展动力。以产业集聚区和新型农村社区建设为载体，加大支持引导力度，城乡统筹发展成效明显，获得了中央及省领导的充分肯定和主流媒体的高度关注。新型农村社区建设持续推进。已启动建设252个、投资59.5亿元，完成建筑面积710万平方米，在建92万平方米，入驻农户2.5万户，拆除旧宅基1.09万亩，复耕或恢复生态966亩，拉动农民住房消费54亿元。产业集聚区建设不断提速。28个产业集聚区的总体发展规划和区域规划环评初步完成。全年完成基础设施投资96亿元，建成区面积已达100平方公里，新入园企业546家，实现规模以上工业总产值935亿元。平原新区基础设施建设加快推进，“十路一桥”全面展开。县域经济稳步壮大。8个县（市）生产总值占全市经济总量的72.9%，比上年提高1.7个百分点；5个县（市）财政一般预算收入超2亿元，其中辉县市、新乡县分别超过9亿元和5亿元。城镇化率达40.96%，比上年提高1.8个百分点。农民转移就业进程加快。共完成农村劳动力转移就业117万人，实现劳务收入70.1亿元，其中42万农民在产业集聚区转移就业。

（六）切实改善民生，构建和谐社会。坚持把保障和改善民生作为应对经济危机的落脚点，完善措施，增加投入，省市两级承诺的20件实事全面完成。就业形势基本稳定。组织172场大型招聘会，为市民提供就业岗位5.5万个，应届毕业生就业率达到82.4%；举办102场农民工专场招聘会，使19万返乡农民工实现了就业。多渠道开发岗位，突出解决“4050”人员就业困难，7700人实现再就业，就业安置率达到88.5％。城镇新增就业15.5万人，下岗失业人员再就业6.2万人，城镇登记失业率为3.7%。发放创业小额贷款1.6亿元，创建省级创业基地7家、国家级创业示范县1个。社会保障力度加大。城镇企业养老保险参保职工总数达61.3万人，新农合参合率达99.2%，均创历史新高。企业离退休职工月人均基本养老金超千元。辉县市被确定为国家首批新型农村社会养老保险试点，参保率居全省县级第1位。新增农村低保对象5万人，城市低保对象减少2万人，共有29.1万人享受低保政策，全年发放低保资金3.4亿元，基本实现了应保尽保、应退尽退。大力推进城镇居民医疗保险，全年参保71.2万人。按政策落实了1.7万多名破产企业退休人员的医疗保险待遇。拓展城乡医疗救助，全年救助困难群众24.8万人，支出资金2072万元。加强保障性住房建设，新开工经济适用房、廉租房25.7万平方米和42.8万平方米；发放廉租住房租赁补贴1349万元，9730户低收入家庭受益。城市环境明显改观。建成“一湖、两线、三园”精品工程，“十路两桥”建成通车，新增绿地面积194万平方米，完成300余座建筑物夜景亮化。10个旧城改造项目完成拆迁任务，市区“城中村”改造累计拆除94.8万平方米。重点城建项目及平原博物院、青少年活动中心等大型公共服务项目加快建设。深入开展全国环保模范城、文明城市、健康城市创建活动，城市品位、市民素质不断提升。社会事业扎实推进。优化教育资源配置，提高薄弱学校办学水平；加强师资队伍建设，提高农村教师素质。职业技术教育资源整合取得重大进展，发展态势良好，位居全省6个职业教育攻坚先进省辖市之首。启动医药卫生体制改革，美国双蓝公司医疗保障合作项目开始运行。甲型H1N1流感、手足口病得到较好防控。人口自然增长率4.94‰，出生人口性别比升高势头得到遏制。新闻出版、防震减灾、民族宗教、体育、统计、外事、侨务、史志、档案、气象、测绘、人防等工作都取得了新的成绩。荣获省“双拥模范城”六连冠称号。社会大局和谐稳定。可防性案事件下降幅度居全省第一，公众安全感指数居全省第二，获得河南平安建设先进省辖市四连冠，红旗区被评为全国和谐社区建设先进城区。信访总量逐月下降，顺利退出全省前五位。深入开展食品安全专项整治，群众健康安全水平不断提升。成为全省唯一的安全城市创建试点市，各类安全生产事故死亡率下降44%。

（七）强化自身建设，保障工作落实。深入开展学习实践科学发展观活动，推进“两转两提”工作，政务水平进一步提升。高度重视权力运行，加强对重点岗位的监督，科室季评机制不断健全。抓好公共资源交易平台建设，进入中心交易项目1142项，增收节支5.8亿元。稳妥推进政府机构改革，精简机构，理顺职能。推进机关“阳光建设”，国务院政务公开办公室充分肯定了我市的做法和成效。强化国资监管和审计监督，提高财政资金使用效益。坚持依法行政，民主法制工作扎实推进。狠抓党风廉政建设，努力建设廉洁政府。

各位代表，面对去年前所未有的困难和挑战，全市上下信心坚定、行动迅速、应对得当、措施有力，在应对危机中加快了发展、增强了后劲、提升了质量、彰显了亮点，许多单项工作位居全省先进行列，在全省十余次重要会议上都作了典型发言。尤为可喜的是，我市三项重点工作进入了全省决策层面：被确定为全省统筹城乡改革发展试验区，平原新区成为全省六大新区之一，动力电池及电动车产业被确定为全省战略先导产业予以优先发展。新乡整体形象进一步提升，荣获全国社会治安综合治理优秀市称号，并被确定为国家知识产权示范创建城市、全国基层农技推广运行机制创新试点市、国家级创建创业型城市试点市。市庆60周年活动的成功举办，国家部委及省内外兄弟单位纷纷来新考察，有力地提升了新乡的知名度，使我们倍受鼓舞、信心大增，进一步激发了全市人民热爱家乡、建设新乡的豪情。一年来的实践再次证明，我市各级领导班子是坚强有力的，广大干部群众、广大企业是能打硬仗、经得起考验的，新乡的未来充满希望。目前，我市发展已站在了一个新的台阶之上，各项成绩来之不易，这是省委、省政府和市委正确领导的结果，是市人大、市政协监督支持的结果，是全市人民团结奋进的结果。在此，我代表市人民政府，向各位人大代表和政协委员，向全市各条战线上的广大工人、农民、知识分子、干部、驻新解放军指战员、武警官兵、公安干警及社会各界人士致以崇高的敬意！向关心、支持新乡发展的朋友们表示衷心的感谢！

同时，我们也清醒地认识到，全市经济社会发展中还存在不少突出矛盾和问题。一是结构调整任务艰巨。产业层次不高，产业集群分工协作程度低，规模大的龙头企业少且辐射带动作用不强。农业品牌产品和产业化龙头企业少，农民持续增收机制尚未健全，年度收入目标没有完成。第三产业占GDP比重偏低，已成为我市经济发展的软肋。进出口总量下降，出口拉动作用仍需增强。二是生态压力依然较大。出境断面水质稳定达标难度较大，地下水资源严重超采，北部山区粉尘排放企业还需要深度治理。三是部分群众就业、生活困难，社会事业发展任务繁重。就业再就业、社会保障、困难企业职工安置压力很大，养老保险基金缺口较大，农村公共服务滞后，社会事业发展与群众需求还有较大差距。四是财政实力弱，市本级和部分县收支缺口较大，预算安排困难。五是化解社会矛盾、处理各种利益诉求、维护社会稳定的任务还比较艰巨。六是政府自身建设还需加强。工作落实力度仍需加大，发展环境需要进一步优化。少数公务人员服务意识淡薄，办事效率不高；个别公务人员奢侈浪费、以权谋私、贪污腐败的问题仍时有发生。对此，我们将认真对待，切实解决。

二、2010年政府工作的指导思想和奋斗目标

2010年是完成“十一五”规划的冲刺之年，也是谋划 “十二五”规划的奠基之年，做好今年工作至关重要。目前全球宏观经济形势复杂多变，经济危机深层次矛盾依然不少，国际贸易保护趋势加剧，经济回升态势仍不稳固，我们必须保持清醒头脑，决不能盲目乐观。同时，我们更要看到诸多机遇，如国家经济结构调整力度加大，我市的一些优势产业属于国家重点扶持范围，这有利于我市产业结构的持续优化升级；国家扩大内需政策效应的日益显现，有利于我市发挥加工工业优势，拓展市场份额，提升服务业水平；地处中原城市群紧密层，中原崛起步伐加快，有利于我市发挥区位优势，扩大招商引资规模。总的看，机遇大于挑战。我们应当坚定信心，主动应对，抢抓机遇，勇于进取，奋力开创新乡跨越发展的新局面。

今年工作的指导思想是：深入贯彻科学发展观，坚持重在持续、重在提升、重在统筹、重在为民，围绕“调整结构、突出转型、加快发展”的总体要求，以统筹城乡发展为主线，以圆满完成“十一五”规划为目标，坚持实施引资项目双带动战略，强力推进“六项行动计划”，统筹推进新型工业化、新型城镇化和农业现代化，调整经济结构，转变发展方式，着力改善民生，保持社会稳定，加快建设效益新乡、创新新乡、生态新乡、和谐新乡，努力实现晋位升级，为综合实力迈向全省第二方阵前列奠定坚实基础。

主要预期目标：生产总值增长12%，其中一、二、三产业增加值分别增长3.8%、15%和11.5%；全社会固定资产投资增长20%，其中城镇固定资产投资增长21%；地方财政一般预算收入增长13%；社会消费品零售总额增长15%；居民消费价格指数涨幅控制在3%左右；城镇居民人均可支配收入、农民人均纯收入均增长8%；城镇新增就业7万人，城镇登记失业率控制在4.5 %以内；人口自然增长率控制在6.4‰以内；单位生产总值能耗降低3.12%；化学需氧量、二氧化硫排放量控制在6.02万吨和5.5万吨；城镇化率提高1.5个百分点以上。

做好全年工作，必须始终把加快发展作为第一要务，在发展中求转变、在发展中求调整、在发展中求提升，用发展的办法解决各种矛盾和问题，保持经济平稳较快增长；必须狠抓结构调整，转变发展方式，推进自主创新和节能减排，提升发展质量和经济效益；必须突出重点，紧紧抓住事关全局、事关长远和人民群众要求最迫切的事情，毫不动摇，持之以恒地加以推进；必须坚持改善民生，善于换位思考，站在人民群众的立场去思考发展、决策工作、处理问题，多做雪中送炭、排忧解难的事情，努力提升群众生活水平；必须转变作风，倡导实干，狠抓落实，务求实效。

三、调整优化产业结构，促进经济跨越发展

产业结构直接影响着发展的速度和质量，我们必须把产业结构的优化升级放在重要位置，切实抓紧抓好。

（一）继续保持投资较快增长。狠抓项目建设，确保城镇固定资产投资突破千亿大关。强力招商引资。注重抓好产业链招商、小分队招商、以商招商和委托招商，重点督查项目开工率和竣工率。深化与中航集团、中兵集团、中铁集团、中建集团、中交股份、中粮集团、中信集团、中纺集团、国家电网等中央企业的战略合作。积极承接沿海产业转移，加强对东南亚、港澳台地区的招商工作。拓宽融资渠道，引进创业投资和风险投资基金，到民间资本活跃地区开展专项招商。不断优化投资环境，增强投资吸引力。全年新批外商投资企业20家，实际利用外资和国内资金分别增长15%、20%。认真落实鼓励出口政策，努力扭转出口下滑局面。加快项目建设。抓好省市重点项目建设，争取进入省经济发展转型升级“双千”规划的项目数量居全省前列。加大中航高科技产业园、河南中兵光电产业园等100个重大工业项目投资力度，确保新乡化纤4万吨短丝等50个2亿元以上项目竣工投产，河南煤化20万吨乙二醇等30个投资5亿元以上的项目全面开工。优化投资结构。围绕 “一谷五基地”六大战略支撑产业、现代服务业、高效农业以及带动作用明显的新兴产业，实施一批投资规模大、科技含量高的转型升级项目，不断提高工业投资、技术改造投资、高新技术产业投资在投资总量中的比重。

（二）促进工业结构优化升级。加大工业结构调整力度，加快新型工业化进程。年内规模以上工业增加值增长18%，销售收入和利润增幅均达20%以上。强力推动“一谷五基地”战略支撑产业发展。集中政府可控资源，设立专项发展基金，壮大产业集群，倾力扶持六大产业发展，构建支撑新乡跨越发展的优势产业体系。制冷产业要重点引进终端龙头企业，发展空调、压缩机、温控装置等产品，扩大冷藏车、冰箱冰柜产能，培植新飞、金龙、新航、科隆、海宝等优势企业集团。生物与新医药产业要努力提高企业创新能力，扩大产业规模，壮大华兰、绿源、双鹭、新谊、飘安等龙头企业，支持金银花深度开发，鼓励华星、拓新等公司开发青霉素、核苷酸等下游产品，全力打造国家级生物医药产业基地。动力电池及电动车产业要尽快突破电池和动力总成关键技术，巩固、扩大电池材料及动力电池产品优势，引进电动车项目，争取上半年市区电动车开始示范运营。特色装备制造业重点支持起重、振动等优势产品发展，提高石化、环保装备制造能力，做好印刷、风电设备等新品开发。煤化工产业要大力发展乙二醇、烯烃等新型高端产品，加快煤层气开发，提高尿素等传统产品的科技含量和附加值。汽车零部件产业要重点抓好中航基地和新飞专用车、汽车电子等项目，谋划建设包括电机、动力总成、充电装备等在内的全国电动车零部件产品基地。六大战略支撑产业年内实现总产值1100亿元，力争三年内取得重大突破，使全市工业规模在3－4年内再翻一番。同时，改造提升化纤纺织、食品、造纸、建材、能源电力等五大传统优势产业，年内工业产值突破600亿元。推动企业重组与协作。加快推进与省电网公司合作建立电动车加电站、中航集团与新乡企业重组、金龙集团与台湾必翔战略合作、中兵集团重组万象公司、中兵集团674厂重组新北仪公司、晋煤集团与延化公司合作，积极推动环宇集团与奇瑞公司合作生产电动汽车动力总成、振华港机与新乡起重企业重组、新飞集团节能照明产业链等战略重组项目，争取早日签约。狠抓节能减排。发展循环经济和低碳经济，积极创建全国环保模范城。规范矿产开发及运管秩序，开展水环境综合整治和太行山生态修复工程。鼓励节能服务产业加快发展，实施十大重点节能工程，淘汰落后工艺和产能。加强执法监管，健全节能减排长效机制。

（三）大力发展品牌农业、高端农业。加快科技兴农步伐，推进“三基地一中心”建设，创名牌、求高效，促进农业增效农民增收，努力把新乡农业打造成全国亮点。夯实农业发展基础。加强农田水利基础设施建设，加快全省现代灌区示范区建设，争取开工建设2处引黄调蓄工程。建设100万亩优质麦种子基地，深耕深松面积100万亩以上。抓好农业综合开发，确保稳产高产，力争粮食总产达到80亿斤以上。扩大园林、花卉、水果、蔬菜等经济作物及高效特色农业面积，大力发展畜牧养殖业。完善科研流通体系。加快中国农科院新乡县万亩试验基地、中科院封丘县高产开发工程、省农科院原阳现代农业示范基地等项目建设。巩固完善38个区域农技服务站，加快基层农技推广运行机制创新试点市建设，引导各地与国家、省、市科研单位建立紧密的战略合作关系，对接各类项目，加快建设现代农业示范园区。依托中粮集团粮食仓储物流基地、中部农产品现货交易中心等重点农业流通项目，构建区域性现代粮食物流中心。加快推进农业产业化。引进全国知名农产品加工企业，支持农业龙头企业发展。年内培育认定市级以上农业产业化龙头企业20家，农民专业合作组织覆盖农户30%以上。发展粮油及瓜果、蔬菜、中药材、食用菌深加工，争创中国名牌农产品，打造品牌农业和高端农业，提升农业经济效益。继续抓好扶贫开发，做好移民安置工作。

（四）努力扩大消费需求。提升服务业总量，发展现代服务业，促进消费，拉动经济增长。加快发展商贸服务业。抓好市级商业中心、区域商业中心、特色商业街和社区商业服务网点建设，培育生活服务型消费市场和新型业态。提升金融服务水平，发展消费型信贷。积极打造文化旅游业。建设重点文化工程，积极发展文化产业。加快建设长垣食博园。加强旅游基础设施建设与管理，建设万仙山国家攀岩公园，全年实现国内旅游收入65.6亿元，接待游客1200万人次以上。着力扩大消费需求。培育新兴消费，推动消费结构升级。规范房地产市场开发，打击囤地行为，促进健康发展，全年新开工商品房230万平方米，完成投资70亿元。推进供销系统经营、组织与服务创新，完善已建成的农村连锁超市，抓好家电、汽车摩托车下乡工作，扩大农村消费。加快城乡消费设施和服务体系建设，强化商品质量监管，净化市场环境，让群众放心消费、方便消费。

（五）加快改革创新步伐。坚持解放思想、改革创新，增强经济社会发展的内生动力。完善经济运行机制。强化经济运行监测，将企业服务年工作措施长效化。支持新乡银行战略重组、扩大规模、跨区域经营。加大资产整合力度，壮大投资集团，争取企业债券发行成功。组建农村商业银行，设立新乡县、辉县市村镇银行。吸引金融机构增设网点，加强对中小企业和“三农”的信贷支持。健全担保体系，筹建担保集团。深入推进各项改革。上半年做好市属国有企业破产、改制收尾工作，不断完善国有资产管理体制。完成市、县政府机构改革，推进事业单位分类改革，完善财政资金投入机制。加快林权、农村综合配套以及文化教育、医药卫生、事业单位养老保险等方面的改革。加快科技创新。新组建省级企业研发中心5家、市级工程技术研究中心20家。组织实施省级重大科技专项5项，力争华兰系列疫苗、绿源药业半合抗生素产业化等项目研发实现新突破。高新技术产业增加值的比重达30%以上，保持全省领先。出台优惠政策，积极引进电池电动车、生物医药、新型煤化工、平原新区建设与管理等方面的高层次专业人才。做好白鹭化纤等10家省级“两化”融合试点单位的信息化项目建设。加强知识产权保护，发明专利占年度专利申请量20%以上。实施名牌战略。争创中国名牌产品2个、中国驰名商标1个、河南省名牌产品6个、省优质产品10个。

四、统筹城乡发展，提升城镇化水平

以新型农村社区和产业集聚区为载体，坚持以工促农、以城带乡，产城一体、产城融合，加快全省统筹城乡改革发展试验区建设，探索积累更多经验。

（一）积极稳妥推进新型农村社区建设。坚持“规划先行、就业为本、农民自愿、因地制宜、量力而行”的原则，利用好相关政策，努力扩大成果。坚定发展方向。建设新型农村社区，符合统筹城乡发展要求，符合群众意愿，方向正确，必须坚持并不断完善。年内要完成全市新型农村社区详规和被整合村庄控制性规划，落实国家建材下乡政策和市里各项扶持政策，抓好重点区域和36个重点乡（镇）新型农村社区建设，完成建房面积550万平方米，同时做好拆除旧宅基、复耕土地等工作。强化就业支撑。依托重点镇和产业集聚区，大力发展二、三产业，广泛吸纳安置当地农民转移就业，增加农民收入。完善公共服务。加快新型农村社区教育、文化、卫生设施建设，建立城乡衔接的公共交通、供水供电、邮政电信、广播电视等公共服务设施体系。重视林业生态建设，健全保洁长效机制，提高农民生活质量。坚持分类推进。注重政府扶持、群众自愿、示范带动、分类指导，在条件成熟的地方加快推进，暂不具备条件的地方要搞好规划，积极创造条件，争取早日实施。要依法依规用地，严禁变相搞房地产开发或圈占耕地。不断改进工作方式方法，切实把好事办好，让农民满意。

（二）加快产业集聚区建设。按照省里要求，加快各项建设，开展升级竞赛，年内争取3-5个产业集聚区进入全省前50强，2-3个新纳入省规划范围。完善发展规划。把产业集聚区规划与土地利用总体规划、城市总体规划有机衔接起来，明确功能定位，选好主导产业，促进产业集聚区健康发展。理顺运行机制。产业集聚区管理机制应统筹、精简、高效。加快市、县两级产业集聚区投融资平台建设，多渠道筹集发展基金。利用好省里电价、用地、异地投资分享税收、区域环评、自主创新等方面的政策，落实我市的扶持措施，推动集聚区加快发展。健全基础功能。坚持高起点、高标准、适度超前的原则，率先完成基础设施建设，同步规划建设生产性、生活性服务设施，着力打造检验检测、信息网络、职业培训等公共服务平台，为招商引资和项目建设创造良好环境。抓好项目引进。注重项目引进质量、产业关联度和环境保护，突出产业特色，提高投资强度，节约土地资源。尽快引进一批龙头企业，促进配套中小企业集聚发展，培育壮大主导产业。纳入省规划范围的产业集聚区全年至少引进10个千万元以上项目，其他产业集聚区至少引进5个千万元以上项目。突出抓好平原新区建设。坚持农业硅谷、科技新城、高新技术产业基地、生态休闲水乡的新区定位，科学编制平原新区总体规划和专项规划，加强与泛华集团合作，完成起步区30平方公里范围内的基础设施主体工程，同步建设公共服务及景观设施。加快建设河南省科研生产试验基地、中部农产品现货交易中心，做好电动车、生物医药、奥特莱斯品牌店、黄河书画院等项目的前期洽谈，争取更多的高科技、高效益项目落户。推动平原新区早出形象、早见效益，形成产业名片，打造全市新的经济增长极。

（三）加强城市建设与管理。巩固城市创建成果，完善城市基础设施，健全城管长效机制，着力提升城市品位，建设生态宜居城市。完善城市功能。搞好县乡公路升级改造，基本完成大外环建设任务，改造建设8条快速通道，建成11条市政道路和3座市内桥梁。确保牧野湖二期、卫河治污疏浚和赵定排环境整治等水系景观工程完工。加快污水处理厂升级改造，启动市区水源地项目。加快市区生活服务设施、游园绿地及生态园区建设，创造良好的生活、休闲环境。加强城市管理，完成“数字城管”向县（市）、区和责任单位延伸的一期工程。抓好新区建设和“城中村”、旧城改造。市区新区核心区建设项目要限定工期，全力推进。平原博物院“五·一”前投入使用，青少年活动中心“三合一”项目年内投用，艺术中心一季度开工建设。将城市危旧房改造、城中村改造与经济适用房及廉租住房建设、城市建设用地挖潜相结合，加大市区西部改造开发力度，建设功能完善、生活便利、环境优美的城市社区。启动军用直升机场搬迁工作，争取上级早日批准，积极推进实施。加快城镇化进程。激活社会资金，提升城镇发展水平和综合承载能力，走城镇带动发展之路。继续开展“新城杯”竞赛，强力推进县城和建制镇的基础设施建设，提高规划和建设管理水平，促进县城面貌进一步改观。

五、着力改善民生，促进社会和谐

坚持着力民生、着力民心，倾听民意，为民解忧，在改善民生方面要舍得投入，加大力度，多办实事。

（一）狠抓就业工作。积极创建国家级创业型城市，确保年内达标。完善全民创业政策体系，提升高校毕业生就业创业能力，力争新增个体工商户8000户、民营企业1000家，均增长10%以上，实现创业存活率70%以上。开展“送岗位、送政策、送服务进家入户”活动，健全就业援助计划，确保85％以上的“4050”人员得到就业安置，年内下岗失业人员再就业2.5万人。完善城乡统一的劳动就业政策，加强城乡人力资源市场建设，形成城乡劳动者公平就业制度。重视技能人才培训，扩大培训规模，逐步提升高技能人才、技师和高级技师的比重。组织劳务输出，增创劳务品牌，培育发展30个具有一定规模的中介机构和劳务输出龙头企业，实现农村劳动力转移就业120万人。认真贯彻落实《劳动合同法》，保障劳动者合法权益。

（二）强化社会保障。完善城镇企业职工养老保险制度，做好辉县市新型农村社会养老保险试点工作，鼓励其他县（市、区）在新型农村社区开展农村社会养老保险，并逐步扩大覆盖面。基本实现医疗保险和工伤保险市级统筹，不断提高各项基本保险覆盖率，扩大保险基金规模。进一步扩大门诊慢性病病种范围，逐步将常见病、多发病门诊医疗费用纳入医疗保险支付范围，扩展受益面。稳妥推进城镇居民医疗保险、新农合的同步结算，新农合人均标准提高50元，达到150元。规范城市低保管理，巩固农村低保扩面提标成果，提高五保户集中供养水平。发展慈善事业，拓宽社会救助渠道。筹集专项资金，做好对困难群众和特殊困难职工的帮扶工作，春节前集中开展一次大病医疗救助。指导用人单位建立正常的工资调整机制，不断提高职工收入水平。建成市流浪未成年人救助保护中心，加快建设市儿童福利院和精神病康复医院改扩建项目。解决17万农村人口的饮水安全问题。加强保障性住房建设，推行租售并举，新开工廉租房40万平方米，市区经济适用房货币补贴2000户，对符合条件的城市住房困难家庭实现应保尽保。

（三）积极发展社会事业。不断优化办学环境，加快实施中小学校舍安全工程，促进义务教育均衡发展，逐步缓解择校现象。筹备成立新乡教育基金会和教育投资公司，建立多元化的教育投融资模式。筹建市职业教育局，加快职教园区和实训基地建设，推动长垣博大烹饪学校建设高等职业学院，抓好幼师、卫校升专工作，打造全国知名的职业教育名片。支持驻新高校建设，优化周边环境，不断拓展校地合作领域。加强农村文化大院和乡镇文化站建设，大力发展社区文化、广场文化，支持发展网络媒体、新型广播电视、报网互动、手机报等媒体业务，丰富群众文化生活。加快市区医疗卫生基础设施建设，开工建设市妇幼保健院病房楼、市人民医院外科综合楼、市二院病房楼，建设新区医疗健康科技园区。结合新型农村社区建设规划，新建标准化村卫生室300所。加大疫病预防控制力度，严防重大疫情蔓延。增加城乡公共健身场所，深入开展全民健身运动和健康城市创建工作。提高污水处理厂运营水平，抓好农村生活污水、垃圾固体废物污染整治，实施城乡垃圾无害化处理，减少粉尘排放，逐步提高市区空气质量优良天数比重。落实计划生育利益导向政策，稳定低生育水平，提高出生人口素质。逐步增加灾害救助投入，提高自然灾害应对能力。依法保障妇女、未成年人和残疾人的合法权益，高度重视老龄事业发展及关心下一代工作。推进文明城市创建，抓好民兵预备役建设和双拥工作，切实做好防震减灾、新闻出版、民族宗教、人防、审计、统计、史志、外事、侨务、档案、测绘等其他工作。

（四）切实维护社会和谐稳定。抓住影响社会和谐稳定的源头性、根本性、基础性问题，做好社会矛盾化解、社会管理创新、公正廉洁执法三项重点工作，全面深化平安建设。加强社会治安综合治理，始终保持“严打”高压态势，再夺“全省平安建设先进市”荣誉称号，保持群众安全感指数全省领先。落实信访稳定工作责任，及时合理解决群众反映的问题，认真处理人民内部矛盾，巩固信访工作成果。开展专项治理，确保食品、药品安全，遏制重特大安全生产事故发生。高度重视舆论导向，提高处理群体性事件、突发事件和应对网络媒体焦点报道的能力与水平。

六、坚持求实务实，加强政府建设

转变政府职能，提高行政效能，强化政风建设，切实增强政府的推动力、贯彻力和执行力，为跨越发展提供坚强保障。

一要巩固为民理念。坚持执政为民，注重维护人民群众的长远利益、根本利益，着力解决好老百姓最关心、反映最强烈的热点难点问题，真正做到发展为了人民、发展依靠人民、发展成果由人民共享，让人民群众得到更多实惠。

二要转变政府职能。深入开展“两转两提”，规范权力运行，不断创新管理方式和工作机制。深化政府机构改革，科学划定职责界限，加强社会管理和公共服务，更好地履行经济调节和市场监管职能，营造良好发展环境。优化公共资源配置，统筹财政资金支出，加大对新兴产业发展和民生重点领域的投入力度。

三要转变工作作风。倡导开短会、讲短话，少发文、发短文，多做少说、苦干实干、注重实效。狠抓落实，把各项任务分解到单位和个人，实行责任制，建立工作台帐，对工作落后的地方和单位加强督查，严格责任追究。重要工作完成情况及时向社会公开，促使各级各部门提升工作效率。

四要提升服务水平。增强服务意识，强化效能监察。推进政务公开，加强各级行政服务中心建设，完善便民代理服务机制，推动公共管理、公共服务的信息化和现代化。健全重大事项集体决策、专家咨询、社会公示与听证等制度。

五要严格依法行政。严格落实行政执法责任制，提高执法质量，加强民主法制工作。自觉接受人大法律监督和政协民主监督，认真办理人大代表、政协委员的议案、提案和建议。广泛听取社会各界的意见，充分发挥群团组织和社会团体的桥梁纽带作用，主动接受社会监督，正确运用群众评议结果，不断改进政府工作。

六要坚持从严治政。牢记“两个务必”，严控支出，勤俭办事，厉行节约，杜绝浪费。认真落实党风廉政建设责任制，狠抓源头治理和制度建设，狠刹损害群众利益的不正之风，严惩违法犯罪的腐败分子，建设廉洁政府。

各位代表！新的一年，各项工作任务艰巨繁重，面对全市人民的殷切期盼，我们既满怀信心，又深感责任重大。让我们深入贯彻落实科学发展观，振奋精神，克难攻坚，为新乡人民群众的幸福生活、为建设中原城市群强市而努力奋斗！
